# The relationship between myocardial microstructure and strain in chronic infarction using cardiovascular magnetic resonance diffusion tensor imaging and feature tracking

**DOI:** 10.1186/s12968-022-00892-y

**Published:** 2022-11-24

**Authors:** N. Sharrack, A. Das, C. Kelly, I. Teh, C. T. Stoeck, S. Kozerke, P. P. Swoboda, J. P. Greenwood, S. Plein, J. E. Schneider, E. Dall’Armellina

**Affiliations:** 1 Biomedical Imaging Sciences Department, Leeds Institute for Cardiovascular and Metabolic Medicine, University of Leeds, Leeds, UK; 2 Institute for Biomedical Engineering, University and ETH Zurich, Zurich, Switzerland; 3 Centre for Surgical Research, University of Zurich and University Hospital Zurich, Zurich, Switzerland

## Abstract

**Background:**

Cardiac diffusion tensor imaging (cDTI) using cardiovascular magnetic resonance (CMR) is a novel technique for the non-invasive assessment of myocardial microstructure. Previous studies have shown myocardial infarction to result in loss of sheetlet angularity, derived by reduced secondary eigenvector (E2A) and reduction in subendocardial cardiomyocytes, evidenced by loss of myocytes with right-handed orientation (RHM) on helix angle (HA) maps. Myocardial strain assessed using feature tracking-CMR (FT-CMR) is a sensitive marker of sub-clinical myocardial dysfunction. We sought to explore the relationship between these two techniques (strain and cDTI) in patients at 3 months following ST-elevation MI (STEMI).

**Methods:**

32 patients (F = 28, 60 ± 10 years) underwent 3T CMR three months after STEMI (mean interval 105 ± 17 days) with second order motion compensated (M2), free-breathing spin echo cDTI, cine gradient echo and late gadolinium enhancement (LGE) imaging. HA maps divided into left-handed HA (LHM, − 90 < HA < − 30), circumferential HA (CM, − 30° < HA < 30°), and right-handed HA (RHM, 30° < HA < 90°) were reported as relative proportions. Global and segmental analysis was undertaken.

**Results:**

Mean left ventricular ejection fraction (LVEF) was 44 ± 10% with a mean infarct size of 18 ± 12 g and a mean infarct segment LGE enhancement of 66 ± 21%**.** Mean global radial strain was 19 ± 6, mean global circumferential strain was − 13 ± − 3 and mean global longitudinal strain was − 10 ± − 3. Global and segmental radial strain correlated significantly with E2A in infarcted segments (p = 0.002, p = 0.011). Both global and segmental longitudinal strain correlated with RHM of infarcted segments on HA maps (p < 0.001, p = 0.003). Mean Diffusivity (MD) correlated significantly with the global infarct size (p < 0.008). When patients were categorised according to LVEF (reduced, mid-range and preserved), all cDTI parameters differed significantly between the three groups.

**Conclusion:**

Change in sheetlet orientation assessed using E2A from cDTI correlates with impaired radial strain. Segments with fewer subendocardial cardiomyocytes, evidenced by a lower proportion of myocytes with right-handed orientation on HA maps, show impaired longitudinal strain. Infarct segment enhancement correlates significantly with E2A and RHM. Our data has demonstrated a link between myocardial microstructure and contractility following myocardial infarction, suggesting a potential role for CMR cDTI to clinically relevant functional impact.

## Background

**Fig. 1:**
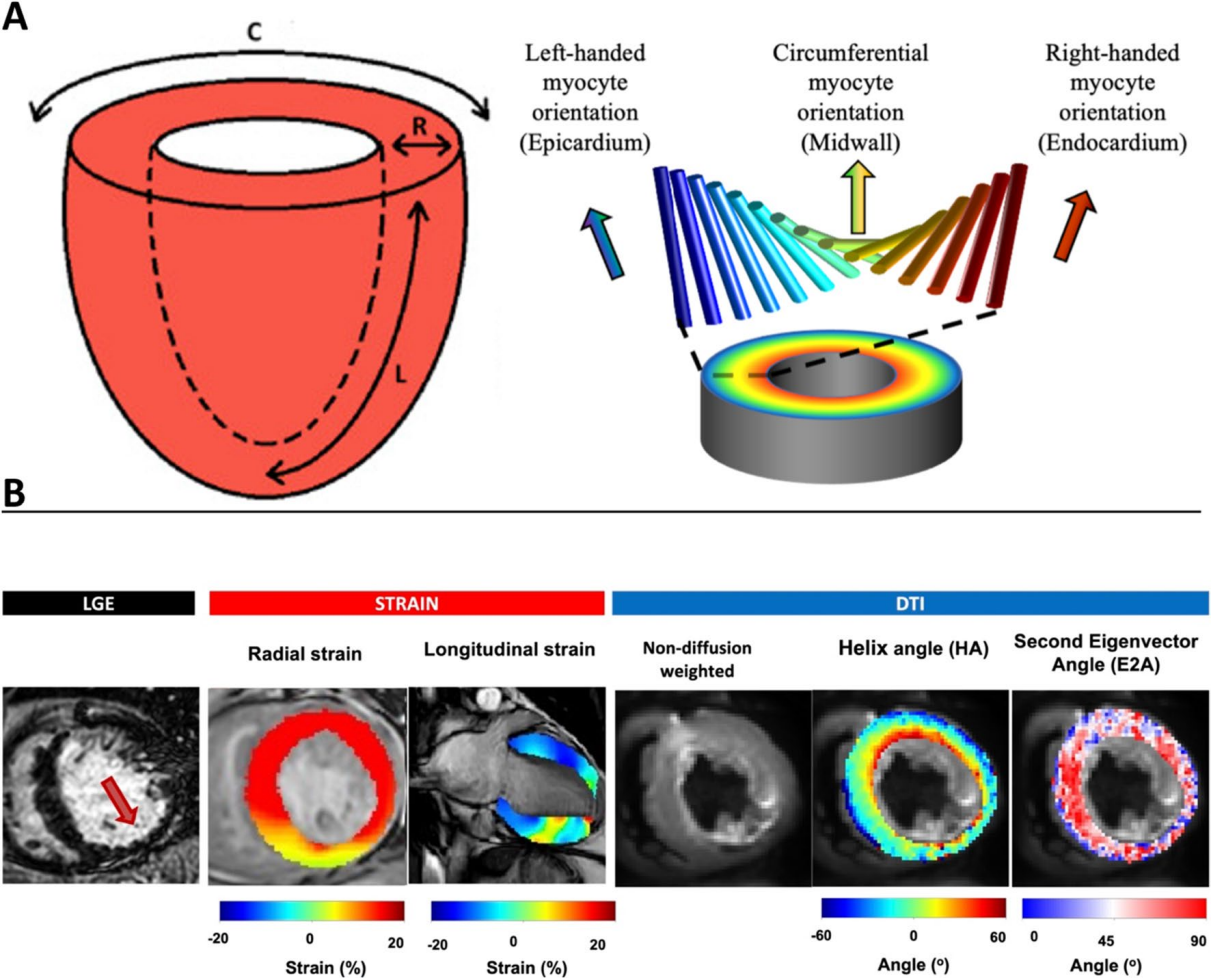
**A** Shows directions of strain in the left ventricle (LV). *C* Circumferential strain, *R* radial strain, *L* longitudinal strain. Helix angle (HA) map showing arrangement of myocytes from left-handed orientation in the epicardium, to circumferential orientation in the mid-wall to right-handed orientation in the endocardium. **B** Case of inferior ST segment elevation myocardial infarctio (STEMI). A subendocardial scar is shown on the late gadolinium enhancement (LGE) short axis image (arrow) with reduced radial and longitudinal strain in the corresponding area of scar. The cardiac diffusion tensor imaging (cDTI) images show a loss of right handed orientation (RHM) on the HA map and a loss of second eigenvector (E2A) in the same scarred mid inferior segment. Adapted from “perioperative clinical utility of myocardial deformation imaging: a narrative review” by E Abuelkasem, 2019, Br J Anaeth, 123 (4):408-420. Copyright 2019 with permission from Elsevier and “acute microstructural changes after ST segment elevation myocardial infarction assessed with diffusion tensor imaging” by A Das, 2021, Radiology, 299(1): 86-96. Copyright 2021 with permission from RSNA

In the healthy heart, cardiomyocytes are arranged in interconnecting helices that transition gradually from left-handed orientation (LHM) in the subepicardium, to circumferential in the mid wall and right-handed orientation (RHM) in the subendocardium. [1–4] (see Fig. 1A). Cardiomyocytes aggregate in laminar secondary structures, several cells thick, known as sheetlets. [5–7] This unique structure allows for the specific ventricular properties of torsion, strain, stress [8, 9] and structural remodelling [10, 11].

Diffusion tensor imaging (DTI) is a cardiovascular magnetic resonance (CMR) based method that allows the non-invasive characterisation of three-dimensional (3D) microstructures in vivo. [12–14] In the heart, cardiac diffusion tensor imaging (cDTI) uses the diffusion of water in the myocardium as an endogenous contrast mechanism. [1, 14–20] Based on the principle that water diffusion occurs preferentially along the long axis of cardiomyocytes, cDTI can provide information on the principal orientations of cardiomyocytes and sheetlets within the myocardium. [21] In cDTI, the secondary eigenvector angle (E2A), reflects sheetlet orientation. The reorientation of sheetlets in the myocardium contributes to myocardial thickening during cardiac contraction and is reflected in the change from low absolute E2A in diastole to high absolute E2A in systole. [22, 23] The mean diffusivity (MD) of water molecules reflects the magnitude of diffusion in a given voxel and the redistribution of intracellular and extracellular space volumes. Fractional anisotropy (FA) measures the directional variability of diffusion in a given voxel. [24] cDTI also allows in-vivo characterisation of the helical arrangement of the cardiomyocytes as validated by dissection plates. [2] The helix angle (HA) is a measure of the elevation angle of the primary eigenvector of the diffusion tensor, corresponding to the long-axis orientation of local cardiomyocytes, with respect to the short axis plane. [25]

In the context of myocardial infarction (MI), these cDTI markers offer exciting opportunities to study acute tissue injury as well as remodelling and a small number of studies have shown promising initial results. Wu et al. demonstrated infarct segments to exhibit a reduction in RHM post-MI, pointing to a loss of organisation amongst subendocardial myocytes. [26] Das et al. confirmed that acutely infarcted myocardium had lower E2A and reduced proportions of RHM corresponding to preferential injury of the sub-endocardium. [27]

There has been limited work looking at the effect of infarct characteristics by cDTI on more detailed cardiac function parameters. [28] CMR feature tracking (CMR-FT) offers an opportunity to quantify myocardial deformation and provide accurate assessment of global and regional circumferential, radial and longitudinal myocardial strain. [29, 30] CMR-FT has been shown to be a superior measure of left ventricular (LV) function and performance early after reperfused MI with incremental prognostic value for mortality over and above LV ejection fraction (LVEF) and infarct size. [31]

We sought to explore the relationship between strain, a sensitive marker of sub-clinical myocardial dysfunction, and cDTI, that allows the non-invasive characterisation of myocardial microstructure in patients at 3 months following ST-elevation MI (STEMI). We propose that cDTI can be used to explain the changes in strain parameters following STEMI and aimed to establish:

1. The relationship between both global and segmental longitudinal strain, and RHM in patients at 3 months post STEMI.
2. The relationship between global and segmental radial strain, and E2A in patients at 3 months following STEMI.
3. The relationship between various DTI parameters (MD, FA, E2A, RHM), infarct size and segmental late gadolinium enhancement (LGE).
4. The relationship between both global and segmental strain and segmental LGE.
5. The relationship between cDTI parameters, and LVEF.

## Methods

### Patient population

Prospectively recruited ‘First-event’ STEMI patients underwent a CMR at 3 months. Study inclusion criteria were *(a)* MI as defined by current international guidelines, [32] *(b)* revascularisation via percutaneous coronary intervention (PCI) within 12 h after onset of symptoms and (c) no contraindications to CMR. Exclusion criteria were (a) previous revascularisation procedure (coronary artery bypass grafts or PCI), (b) known cardiomyopathy, (c) severe valvular heart disease, (d) atrial fibrillation and (e) haemodynamic instability lasting longer than 24 h following PCI and contraindication. The study protocol was approved by the institutional research ethics committee and complied with the Declaration of Helsinki; all patients gave written informed consent for their participation. (NIHR 33963, REC 17/YH/0062).

### Cardiovascular magnetic resonance imaging

CMR was performed on a 3 T scanner (Achieva, Philips Healthcare, Best, The Netherlands) and included: full LV coverage by functional cine and LGE imaging, three matching short-axis slices (located at the base, mid and apex) by cDTI, modified Look-Locker inversion (5(3)3 MOLLI) T1 mapping, T2 mapping and post-contrast T1 mapping as previously described [27]. cDTI data were acquired using electrocardiogram (ECG)-gated second-order motion-compensated single-shot spin echo (SE) echo planar imaging sequence with bipolar M1M2 bipolar diffusion waveforms [33] and respiratory navigator tracking: (TE/TR = 89 ms/3RR intervals, Flip angle = 90°, FOV = 238 × 238 mm, matrix = 108 × 105, acquired in-plane resolution = 2.20 × 2.27, slice gap = 8 mm, reconstructed voxel size = 1.7 × 1.7 × 8 mm, SENSE acceleration = 1.8). A respiratory echo-based navigator was placed on the right hemi-diaphragm with a 50 mm gating window and continuous gating level drift activated. A cylindrical CMR radiofrequency excitation pulse from which a 1-dimensional projection of the lung-liver interface was generated and was used to infer the breathing phase. The navigator was played at the start of the R-R interval, at end-diastole of the cardiac cycle. The steady-state of ongoing balanced steady-state free precession (bSSFP) readout was stopped in the standard controlled manner by using half-alpha radiofrequency pulses to temporarily store the steady state magnetization in the z-direction**.**

Each cDTI dataset constituted 18 non-collinear diffusion-weighted (DW) acquisitions with b-values of 100 s/mm^2^ (× 3), 200 s/mm^2^ (× 3), and 500 s/mm^2^ (× 12) as previously described and validated [34, 35]. We avoid using b = 0 s/mm^2^ to suppress the signal from the blood pool and myocardial perfusion, and we fit the diffusion tensors to all the data points including b = 100, 200 and 500 s/mm^2^, therefore avoiding the need to have a 'reference b-value'.

Based on cine data, trigger delay was set individually for each patient to coincide with 60% peak systole and the centre of k-space was approximately at 85% of peak systole. cDTI acquisition was successful in all patients (mean acquisition time 13 ± 5 min).

### CMR analysis

Cine, strain and LGE data were analysed using cvi42 (version 5.9.4, Circle Cardiovascular Imaging Inc, Calgary, Canada,) to derive LVEF, global strain parameters and infarct size as previously reported [27]. Quantitative assessment of LGE images was performed using a threshold of > 5 standard deviations above remote, normal myocardium. To investigate changes in cDTI parameters in patients with worsening LV function, three groups of patients were identified based on LVEF (in accordance with European Society of Cardiology Guidelines) [36]: Group 1 with preserved LVEF% (pEF, EF > 50%), group 2 with mid-range ejection fraction (mrEF, LVEF 40–49%), and group 3 with reduced ejection fraction, (rEF < 40%).

3D strain was analysed using cine short axis, cine 2 and 4 chamber views to derive global radial strain (GRS), global circumferential strain (GCS), and global longitudinal strain (GLS). Smoothed endocardial and epicardial borders were manually drawn in the end-diastolic frame, (defined as the phase with the largest LV volume), for all short and long axis slices before defining the superior right ventricular (RV) insertion points within the LV. End-diastolic phase had to be identical in all short axis and long axis slices of one subject. LV outflow tract (LVOT) and apical segments, were completely excluded in all short axis slices. Using 3D FT, a 3D deformable model of the myocardium was generated in the end-diastolic phase by interpolating the endo and epicardial boundaries tracked by the 2D algorithm. The accuracy of feature tracking was manually checked following automated strain analysis on the 2D and 3D CMR models by assessing the tracking of the endocardial and epicardial borders. Tracking quality and segmentation was also evaluated using software tools like mesh, boundaries or myocardial points. If contours did not follow the epicardial or endocardial borders correctly, delineation was retraced and adjusted. In cases of remaining tracking issues, those segments were excluded from analysis and not considered for global strain assessment.

The algorithms used by cvi42 to analyse 2D and 3D strain analysis have been previously described and their validity demonstrated by Liu et al. [37]. To summarise, 2D CMR-FT determines myocardial deformation using reference points placed on the mid myocardial wall, which are tracked over the cardiac cycle in the short-axis or long-axis cine images. When the myocardium contracts and relaxes, these reference points move and can be tracked by surrounding features in two directions, therefore giving independent 2D motion fields for short- and long-axis data. By combining the 2D short and long axis image information into a single 3D motion field, a 3D deformation model is generated. Based on the motion fields, the myocardial strain is quantified either globally or segmentally in radial, circumferential and longitudinal directions.

### cDTI post-processing

cDTI data processing was performed using in-house developed MATLAB (Mathworks, Natick, Massachusetts, USA) as described previously. [27] Quality control and assessment of scan quality was undertaken by visual assessment by two experienced investigators (AD) and (CK). CK was blinded to clinical data—this involved subjectively identifying DW images corrupted by artefact or failed registration and omitting them from further processing. After manual data rejection, 10 ± 2 (DW) repetitions were available per diffusion gradient orientation for the construction of averaged DW images and tensor calculation. This was inclusive of base and mid slices only; apical data was excluded from the study due to persistent data quality issues from unsuppressed fat, signal loss and visually appreciable suboptimal signal-to-noise ratio. Based on the registered data, magnitude images were averaged across accepted repetitions, according to diffusion direction and b-value. Tensor eigenvalues, MD, FA, HA, and E2A maps were calculated based on the tensors derived from cDTI data. Endo- and epicardial borders were manually delineated based on the reconstructed non-diffusion weighted data; cine-images in the same phase of the cardiac cycle were used as a visual reference for more precise recognition of borders. Both region-of-interest (ROI) based and segmental analysis were performed as described below.

### Regions of interest (ROI) analysis

ROIs manually drawn in accordance with standards set by the European Association for Cardiovascular Imaging [38] were used for the analysis of MD_(ROI)_ and FA_(ROI)_ in the infarct and remote myocardium, located 180 degrees opposite the infarcted myocardium. To derive accurate DTI measurements of the full infarcted region, a voxel-wise co-registration of LGE and DTI images was needed. As LGE and DTI images are acquired in different phases of the cardiac cycle (diastole vs systole), we performed a visual co-registration to identify the core of the scar. The ROI approach allowed us to be conservative and detect with certainty the core of the scar avoiding including the infarct border zone.

### Segmental analysis

After dividing each slice into 6 equiangular segments starting from the anterior interventricular junction [38], segmental analysis was undertaken to derive: HA DTI markers, segmental radial strain, segmental circumferential strain, segmental longitudinal strain, segmental LGE%. Segmental LGE% (damaged area/ segmental area) refers to the percentage of gadolinium enhancement of a given American Heart Association (AHA) segment. Within the infarcted myocardium, the segment with the maximal LGE% extent (i.e. infarct segments) was identified as representative of the infarct and included in analysis. Infarct segments were grouped as: no LGE; 1–25%, 26–50%, 51–75%, and > 75%.

HA DTI segmental _(SEG)_ markers were described by classifying voxels from HA maps to one of three groups [LHM_(SEG)_ (− 90° ≤ HA < − 30°), CM_(SEG)_ (-30° ≤ HA ≤ 30°) and RHM_(SEG)_ (30° < HA ≤ 90°)] and quantitative markers derived as the respective myocardial proportions of each type as previously described [27]. Absolute E2A values were quoted.

### Inter-observer variability

To assess the interobserver reproducibility of cDTI analysis, all 32 cDTI scans were analysed by two experienced investigators (AD) and (CK). The reproducibility of our cDTI analysis has been previously reported [27, 34].

### Statistical analysis

Statistical analyses were performed in SPSS (version 21.0, Statistical Package for the Social Sciences, International Business Machines, Inc., Armonk, New York). Normality was checked using the Shapiro–Wilk test. Continuous variables are reported as mean ± SD. Comparison between quantitative variables was performed by independent-sample parametric (unpaired Student’s t-test) or non-parametric (Mann–Whitney) statistical test as appropriate. For comparing results from initial and repeated measurements, paired t-tests, and ANOVA with Bonferroni post-hoc comparisons were used. Pearson correlation analysis was used to calculate the correlation coefficient between cDTI and strain as well as LVEF% recovery.

Univariate analyses were performed to identify predictors of reduced LVEF at 3 months. Variables with a probability value < 0.1 in the univariate analysis were included in a multivariable linear regression analysis. Interobserver variability was analysed using the Bland–Altman method. All tests were assumed to be statistically significant when *p* < 0.05.

## Results

### Baseline patient characteristics

Baseline patient characteristics are given in Table 1. Thirty-two patients (M: F = 28:4, age 60 ± 10 yrs), completed CMR examinations three months following their STEMI (105 ± 17 days) and were included in the analysis. The mean LVEF was 44 ± 10% (Table 2): 10 patients (31%) had rEF (LVEF 34 ± 3%), 14 patients (44) had mrEF (LVEF 45 ± 2%), 8 patients (25%) had pEF (LVEF 58 ± 5%). Global infarct size was 18 ± 12 g with mean LV% of 22 ± 11%. Segmental LGE% in infarct segments were 66 ± 21% and distributed as follows: < 25% in 2/32 patients (6%), 26–50% in 5/32 (16%), 51–75% in 13/32 (41%) and 76–100% in 12/32 (38%). One patient had a previous MI in a different territory. In this patient, the remote segment was chosen to exclude both the recent and previous myocardial infarction. Although 20/32 patients had microvascular obstruction (MVO) at first presentation, none had persistent MVO at 3 months.

**Table 1:** Baseline patient characteristics

Patient Characteristics	Value (n = 32)
Age (years)	60 ± 10
Sex (M/F)	28/4
*Risk factors*	
Smoker	7
Hypertension	7
Diabetes	7
Family history	7
Peripheral vascular disease	1
*Characteristics at presentation*	
Culprit Coronary Artery	
Left anterior descending artery	16
Left circumflex artery	3
Right coronary artery	13
Microvascular obstruction	20
Time from onset to balloon inflation time (min)	231 ± 154
*Treatment*	
Aspirin	32
PY2I	32
ACE inhibitor	32
Beta blocker	32

*ACE* angiotensin converting enzyme

**Table 2:** Global CMR findings

Parameter	Mean (n = 32)
Interval from PCI to CMR Time (days)	105 ± 17
LVEF (%)	44 ± 10
LVEDV (ml)	171 ± 35
LVEDVI (ml)	89 ± 17
Mean LGE (% of LV)	22 ± 11
Mean infarct segment enhancement (%)	66 ± 21
Infarct size (g)	18 ± 12
Global radial strain (%)	19 ± 6
Global circumferential strain (%)	− 13 ± 3
Global longitudinal strain (%)	− 10 ± 3

*Normal strain values as assessed by 3D FT CMR are as follows: global radial strain (22–73), global circumferential strain (− 13 to − 23) and global longitudinal strain (− 9 to − 20) [37]. *LGE* late gadolinium enhancement, *LVEDV* left ventricular end-diastolic volume, *LVEDVI* left ventricular end-diastolic volume index, *LVEF* left ventricular ejection fraction

### DTI in chronically infarcted myocardium

**Fig. 2:**
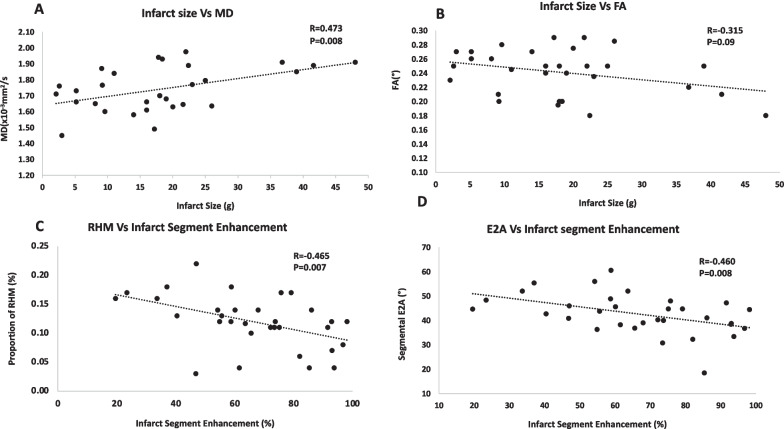
Correlations between cDTI parameters and infarct size and segmental LGE% three months post STEMI. **A**, **B** Show correlation between infarct size and mean diffusivity (MD) and fractional anisotropy (FA), respectively. Panels C and D show correlations between RHM (right-handed myocytes) and E2A (secondary eigenvector) and infarct segment enhancement respectively

MD in infarcted myocardium was significantly higher than in the remote myocardium (MD _infarct ROI_ = 1.74 ± 0.14 × 10^–3^ mm^2^/s vs MD _remote ROI_ = 1.46 ± 0.09 × 10^–3^ mm^2^/s, P = 0.019). FA was lower in infarcted myocardium, compared to the remote segments: FA _infarct ROI_ = 0.24 ± 0.03 vs FA _remote ROI_ myocardium = 0.33 ± 0.03, p = 0.127 (see Table 3). Whilst MD correlated significantly with global infarct size (r = 0.473, p = 0.008), FA showed a negative correlation which did not reach significance (r = − 0.315, p = 0.09, Fig. 2A, B). There was also a significant correlation between both RHM and E2A and segmental LGE% (r = -0.465, p = 0.007, r = − 0.460, p = 0.008, respectively, Fig. 2C, D). MD and FA did not correlate significantly with segmental LGE% (r = 0.218, p = 0.248 and r = 0.047, p = 0.806 respectively).

**Table 3:** CMR findings according to left ventricular ejection fraction

Parameter	pEF (n = 8)	mEF (n = 14)	rEF (n = 10)	ANOVA P Value
LGE _(SEG)_	49 ± 17	66 ± 20	81 ± 16	0.004
Infarct Size	13 ± 8	16 ± 10	28 ± 12	0.011
cDTI				
MD_(ROI)_ (× 10^−3^mm^2^/s)	1.6 ± 0.1	1.7 ± 0.1	1.9 ± 0.1	< 0.001
FA_(ROI)_	0.3 ± 0.2	0.3 ± 0.3	0.2 ± 0.3	0.002
Absolute E2A_(SEG)_ (Degrees)	51 ± 5	43 ± 5	35 ± 7	< 0.001
Helix angles				
RHM_(SEG)_ (%)	17.0 ± 2.6	12.4 ± 3.0	7.6 ± 3.3	< 0.001
CM_(SEG)_ (%)	73.5 ± 7.2	71.1 ± 10.6	69.4 ± 9.4	0.664
LHM_(SEG)_ (%)	8.5 ± 5.2	17.8 ± 10.2	23.6 ± 8.8	0.004
Strain				
Global radial strain (%)	26.3 ± 6.6	17.8 ± 3.4	14.1 ± 3.5	< 0.001
Global longitudinal strain (%)	− 12.9 ± 2.5	− 10.5 ± 2.4	− 7.4 ± 2.6	< 0.001
Global circumferential strain (%)	− 15.7 ± 3.3	− 12.4 ± 1.4	− 11.1 ± 2.9	0.002
Radial strain in infarct segments (%)	16.5 ± 9.6	13.7 ± 6.8	8.5 ± 3.7	0.051
Longitudinal strain in infarct segments (%)	− 13.7 ± 4.1	− 10.1 ± 3.5	− 6.9 ± 2.1	< 0.001
Circumferential strain in infarct segments (%)	− 14.0 ± 7.4	− 8.4 ± 5.4	− 6.8 ± 6.1	0.052

*cDTI* cardiac diffusion tensor imaging, *E2A* secondary eigenvector, *FA* fractional anisotropy, *LHM* left handed orientation, *MD* mean diffusivity, *RHM* right handed orientation

### Strain in chronically infarcted myocardium

Global strain values are shown in Table 2. GRS and GCS values were reduced with preserved GLS. A significant difference was seen between segmental strain values in infarcted and remote segments (Table 3). (The null hypothesis used was that there was no difference between strain and cDTI parameters between remote and infarcted myocardium). There was significant correlation between GRS and segmental LGE% (R = − 0.420, p = 0.017) and between GCS and segmental LGE% (R = 0.389, P = 0.028). There was no significant correlation between GLS and segmental LGE% (R = 0.165, p = 0.368) nor between any global strain marker and infarct size.

### Relationship between strain and cDTI

**Fig. 3:**
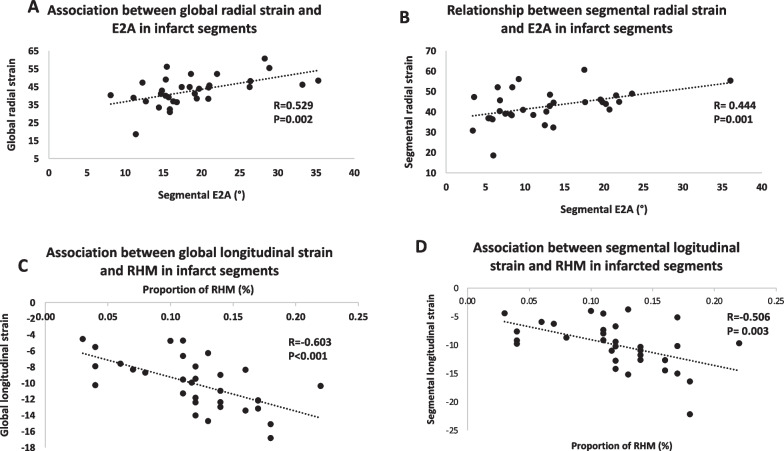
Associations between global and segmental strain parameters and cDTI parameters. Panels A and B show association between global and segmental radial strain and E2A (secondary eigenvector) in infarct segments. Panels C and D show correlations between global and segmental longitudinal strain and RHM (right-handed myocytes) in infarct segments

In chronically infarcted myocardium, correlations were found between: GRS and E2A (R = 0.529, p = 0.002) (Fig. 3A), GLS and proportion of RHM (R = − 0.603, p < 0.001), (Fig. 3C), segmental radial strain and segmental E2A (R = 0.444, p = 0.011) (Fig. 3B), segmental longitudinal strain and the proportion of RHM (R = -0.506, p = 0.003) (Fig. 3D).

Cardiac biomechanics is a complex field where all parameters are related to each other; we report a complete analysis of the relationship between strain and cDTI in the supplement table (see Appendix, Tables 4 and 5). In addition to the above results, Table 4 shows a significant correlation between both global and segmental radial strain and RHM (R = 0.697, p < 0.001 and R = 0.498, p = 0.004, respectively) and between both global and segmental longitudinal strain and E2A (R = − 0.687, P < 0.001 and R = − 0.558 and p = 0.001). Furthermore, whilst circumferential strain has been reported to have prognostic value in STEMI patients, [39], our study shows only a modest correlation with most cDTI parameters but not E2A and RHM. Larger studies will be needed to address this important point. In remote myocardium, the observed correlations were much lower and non-significant compared to infarcted segments. As shown in the supplemental Table 5 (please see Appendix) in the remote segments, E2A did not correlate with radial strain and RHM did not correlate with longitudinal strain.

**Fig. 4:**
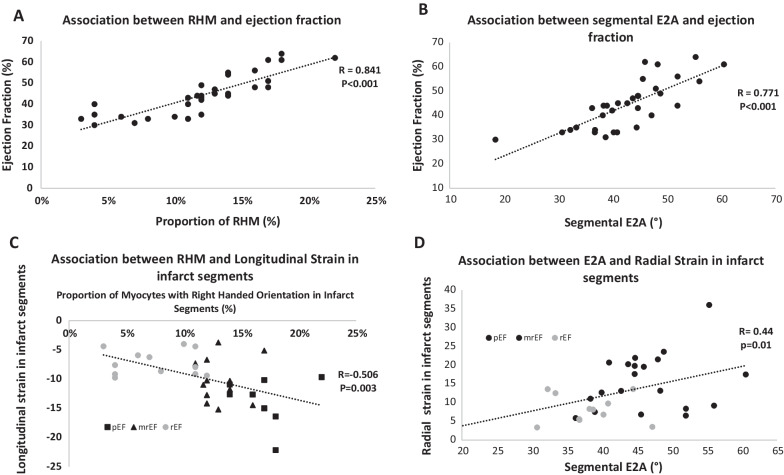
Correlations between right-handed myocytes (RHM), Secondary Eigenvector Angle (E2A), left ventricular ejection fraction (LVEF) and strain. **A** Shows correlations between RHM and LVEF. **B** Shows correlation between E2A and LVEF. **C** Shows correlation between RHM and longitudinal strain divided into LVEF groups. **D** Shows correlation between E2A and radial strain, divided into LVEF groups

To investigate the existing link between myocardial deformation, myocardial structure by cDTI, and LV remodelling post infarct, we compared markers in different groups based on LVEF value. GRS, GCS, and GLS all differed significantly between groups (Table 4). When focusing on segmental strain in infarcted myocardium, the only strain parameter showing a significance difference between LVEF groups was GLS (p < 0.001). On the other hand, cDTI markers assessed in the infarcted myocardium (MD _(INFARCT ROI)_, FA _(INFARCT ROI)_, E2A_(SEG)_ and RHM_(SEG)_) all differed significantly between LVEF groups. E2A_(SEG)_ and RHM_(SEG)_ correlated significantly with LVEF (Fig. 4A, B). Whilst a correlation between strain and cDTI was significant, this was only moderately strong indicating some degree of association (Fig. 4C, D).

## Discussion

The interactions between numerous physiological and biomechanical parameters including myocardial microstructural injury, infarct size, extent and location, strain and global function are immensely complex. Whilst standard CMR imaging modalities allow for quantitative accurate assessment of many of the above crucial parameters, only recently the quantification of microstructural changes using cDTI have become possible. This exploratory study provides new insight into the mechanistic and functional link between myocardial deformation by FT and 3D structure of the myocardium by cDTI in patients with chronic MI. Our main findings are: (a) increasing segmental extent of infarction on LGE is associated with increasing loss of myocytes with RHM by DTI and the orientation of sheetlets measured by E2A; (b) segments with fewer subendocardial cardiomyocytes evidenced by a lower proportion of myocytes with RHM on HA maps show reduced longitudinal strain; (c) the loss of sheetlet orientation assessed using E2A correlates with worsening radial strain; (d) cDTI parameters such as MD correlate well with infarct size in the chronic stage; (e) cDTI parameters in chronic infarct correlate with EF.

### cDTI to detect chronic infarction

Cardiomyocytes are arranged in interconnecting helices that transition from LHM in the subepicardium, to circumferential in the mid wall and RHM in the subepicardium. [1–5] These transmural differences of cardiomyocyte orientation within the myocardial wall can be appreciated non-invasively using cDTI. The pathophysiology of MI is characterised by a progressive ischaemic wave from the subendocardium to the subepicardium. [40] Here we demonstrate an association between cDTI biomarkers and the segmental extent of infarct size. As previously shown [17, 27], MD is expected to increase in infarct zones due to increased extracellular space from cell death causing water diffusion to become less restricted. [28, 41, 42]. Accordingly, we show an increase in MD proportional to the infarct size. Additionally, at a segmental level, E2A (reflecting the loss of sheetlet angularity during systole) and RHM (loss of organisation among subendocardial cardiomyocytes) [22, 42] correlated with the transmural extent of infarction.

### Relationship between cDTI and strain in chronic MI

There is a clear functional and mechanistic link between deformation and 3D structure of the myocardium. Myocardial deformation in patients post MI is impaired with strain values inversely related to infarct size and infarct transmurality [37]. Myocardial strain by FT has incremental prognostic value compared to standard LGE infarct size and EF [31]. Preserved sheetlet angularity and organisation of cardiomyocyte arrangement plays a crucial role in maintaining LV geometry and function [43]. Radial strain is thought to be driven by the dynamic reorientation of sheetlets while longitudinal strain is thought to relate to the subendocardial function [44, 45]. The orientation of sheetlets, E2A, is disrupted in MI [42] and radial strain has been shown to be dependent on the orientation of sheetlets [44, 45]. It therefore follows logically that low E2A, caused by the disruption to laminar sheetlet orientation from MI, corresponds to low radial strain and this is supported by our findings. Wu et al. previously demonstrated infarct segments to exhibit a reduction in RHM post-MI, pointing to a loss of organisation amongst subendocardial myocytes. [26]. This has also been shown more recently by Das et al. [27] where acutely infarcted myocardium had lower E2A and reduced proportions of RHM corresponding to sub-endocardium. Although previous studies have mostly used stimulated echo acquisition mode (STEAM) cDTI [26], single-shot SE cDTI has been proposed as a refined alternative to STEAM, providing higher signal-to-noise ratio and more reproducible images [35] by allowing for free breathing and shorter scan times. [33] A small recent study has combined the assessment of cDTI and strain in pig models following MI. They have shown a significant correlation between longitudinal strain and transmural HA gradient (r = 0.59, P < 0.05) in chronic MI. [28] We have shown that in patients 3 months post STEMI, segments with less subendocardial cardiomyocytes evidenced by a lower proportion of myocytes with RHM on HA maps correlate with worse longitudinal strain. Such association is not observed in remote normal myocardial segments where the 3D microstructure is still preserved at 3 months. Further investigations looking at long term remote DTI changes following LV remodelling will be needed. Since the subendocardium is lined by cardiomyocytes in a RHM, it follows that MI would result in impaired longitudinal strain, and that the larger the MI, the worse the longitudinal strain. Whilst biomechanically speaking, these associations are logical, we cannot oversimplify the relationship between myocardial microstructure and function. Our results investigate even further the existing association between microstructure and deformation by showing how changes in RHM and E2A correlate also with radial and longitudinal strain respectively.

### cDTI and EF

The orientation and organisation of the sheetlets in the myocardium is crucial to maintain an efficient pumping mechanism for the LV by determining optimal myocardial deformation. [22] Previous observations demonstrate that cardiac muscle activity during contraction is not isometric, and early shortening occurs within the subendocardial myofibers in the anterior wall of the LV [46–48]. It therefore follows that any disruption to the orientation of the sheetlets, evidenced by lower E2A and/or reduced proportions of RHM would result in impaired myocardial contraction and therefore impaired strain and LVEF. In our study we noted that cDTI and strain parameters differed significantly between LVEF populations. Panel B in Fig. 1 shows cDTI strain and LGE images in a patient who suffered an inferior STEMI.

### Clinical implications

Although cDTI is unlikely to replace LGE in clinical practice, it could have an important role in clinical applications as a non-contrast method not only to identify acute and chronic scarring [27] but also to relate the extent of damage to the pathophysiological consequences on LV remodelling. The reported observations are a first indication that cDTI can add to the current armamentarium of CMR methods for the assessment of the adverse effects of myocardial infarction in patients with a high procedural success rate and image quality. In addition, our findings indicate that cDTI combined with strain analysis may help explain the structural remodelling and changes that occur following STEMI. The combined use of cDTI and strain assessment provides new insight into the impact of MI on myocardial deformation and may help predict outcomes and likelihood of myocardial recovery. By showing that cDTI and strain parameters differ significantly between LVEF populations at 3 months, risk stratification based on cDTI, and strain may add further incremental prognostic value compared to standard LGE size and LVEF. cDTI might well be suited to increase further the predictive value of FT techniques by reflecting abnormal segmental myocardial deformation due to changes in tissue composition. Future studies will have to determine if such findings can help risk stratify patients.

## Limitations

The interactions between myocardial microstructure and contractility are highly complex and cannot be fully described by cDTI and strain imaging, a limitation ultimately shared with all other in vivo imaging modalities, which can only approximate the physiological and pathophysiological processes in a living organism. Our sample size was relatively small, and a larger study population will be needed to further explore the associations we have found. Furthermore, there are unavoidable problems with strain calculations using FT. For example, radial strain estimates using CMR are often omitted due to limited accuracy and precision since estimation is usually more prone to errors due to image resolution and noise [49] This is seen in our data where we observed reasonably low radial strain measurements in our results. Furthermore, segmental strain values are not commonly used as they have been shown to be less reproducible compared to global strain measurements [50–52] Segmental strain should therefore be interpreted with caution. Furthermore, omission of the apical cDTI slice, due to persistent data quality issues from unsuppressed fat, signal loss and visually appreciable suboptimal signal-to-noise ratio is a frequent limitation of cDTI. Furthermore, despite our best efforts, our MD and FA DTI slices were calculated using ROIs and therefore did not correspond precisely to our infarct AHA segments.

## Conclusion

This study demonstrates the supportive role between strain assessment using FT and cDTI in the assessment of patients with chronic MI. We propose that cDTI may be used as an additional tool to help explain the structural remodelling and changes that occur in the myocardium following STEMI. Although clinical applications of cDTI are yet to be fully validated and established, our results help explain the complex association between myocardial microstructure and regional function. They also suggest the important incremental value of cDTI in the assessment of infarct transmurality which has important clinical implications. However, further larger studies are needed to validate these findings.

## Data Availability

The datasets used and analysed during the current study are available from the corresponding author on reasonable request.

## Appendix

See Tables 4, 5 and 6.

**Table 4:** Correlation table in infarct segments. Correlation is using Pearson correlation. P value is considered significant at the 0.05 level

	RHM	CHM	LHM	FA	MD	E2A
**GLS**						
Correlation	− 0.603	− 0.243	0.572	− 0.661	0.669	− 0.673
P value	< 0.001	0.179	< 0.001	< 0.001	< 0.001	< 0.001
**GRS**						
Correlation	0.697	− 0.018	− 0.371	0.552	− 0.693	0.529
P value	< 0.001	0.922	0.037	0.002	< 0.001	0.002
**GCS**						
Correlation	− 0.608	− 0.144	0.438	− 0.404	0.515	− 0.591
P value	< 0.001	0.431	0.012	0.027	0.004	< 0.001
**SLS**						
Correlation	− 0.506	− 0.193	0.486	− 0.442	0.423	− 0.558
P value	0.003	0.289	0.005	0.015	0.02	0.001
**SRS**						
Correlation	0.498	0.134	− 0.440	0.414	− 0.478	0.444
P value	0.004	0.466	0.012	0.023	0.008	0.011
**SCS**						
Correlation	− 0.466	0.063	0.221	− 0.360	0.394	− 0.341
P value	0.007	0.732	0.224	0.051	0.031	0.056

*SLS* segmental longitudinal strain, *SRS* Segmental radial strain, *SCS* Segmental circumferential strain

**Table 5.** Correlation table in remote segments. Correlation is using Pearson correlation. P value is considered significant at the 0.05 level

	RHM	CHM	LHM	FA	MD	E2A
**GLS**						
Correlation	0.079	− 0.268	0.222	− 0.354	0.537	− 0.023
P value	0.669	0.137	0.222	0.060	0.003	0.901
**GRS**						
Correlation	0.104	0.165	− 0.130	0.406	− 0.522	0.01
P value	0.573	0.368	0.478	0.029	0.004	0.957
**GCS**						
Correlation	− 0.097	− 0.389	0.356	− 0.408	0.408	0.092
P value	0.597	0.028	0.045	0.028	0.028	0.615
**SLS**						
Correlation	0.107	− 0.298	0.249	− 0.221	0.461	− 0.051
P value	0.560	0.098	0.169	0.249	0.012	0.781
**SRS**						
Correlation	0.430	− 0.022	− 0.159	0.222	− 0.260	− 0.058
P value	0.014	0.906	0.385	0.247	0.173	0.752
**SCS**						
Correlation	− 0.189	0.044	0.088	− 0.318	0.145	0.030
P value	0.300	0.810	0.633	0.093	0.452	0.872

*SLS* segmental longitudinal strain, *SRS* Segmental radial strain, *SCS* Segmental circumferential strain

**Table 6:** Correlation table looking at cDTI parameters by infarct location. Correlation is using Pearson correlation. P value is considered significant at the 0.05 level

	Mean ± SD	95% CI	ANOVA p value
**Infarct MD**			0.070
LAD territory	1.79 ± 1.22	1.71–1.84	
LCX territory	1.55 ± 0.14	0.28–2.82	
RCA territory	1.73 ± 0.14	1.64–1.81	
**Infarct FA**			0.552
LAD territory	0.24 ± 0.32	0.22–0.26	
LCX territory	0.27 ± 0.07	0.20–0.33	
RCA territory	0.24 ± 0.03	0.22–0.27	
**Infarct E2A**			0.152
LAD territory	40.14 ± 9.53	35.24–45.04	
LCX territory	48.60 ± 0.35	45.42–51.78	
RCA territory	45.13 ± 5.77	41.64–48.62	
**Infarct Endo**			0.741
LAD territory	0.12 ± 0.05	0.09–0.14	
LCX territory	0.15 ± 0.04	− 0.17–0.46	
RCA territory	0.12 ± 0.04	0.10–0.14	
**Infarct mid**			0.591
LAD territory	0.70 ± 0.09	0.65–0.74	
LCX territory	0.77 ± 0.04	0.45–1.08	
RCA territory	0.72 ± 0.10	0.66–0.78	
**Infarct epi**			0.310
LAD territory	0.19 ± 0.10	0.14–0.25	
LCX territory	0.09 ± 0.00	0.09–0.09	
RCA territory	0.16 ± 0.11	0.09–0.22	

*LAD* left anterior descending coronary artery, *LCX* left circumflex coronary artery, *RCA* right coronary artery

## Abbreviations

3D: Three-dimensional
AHA: American Heart Association
bSSFP: Balanced steady state free precession
cDTI: Cardiac diffusion tensor imaging
CM: Circumferential myocytes
CMR: Cardiovascular magnetic resonance
DW: Diffusion weighted
DTI: Diffusion tensor imaging
E2A: Secondary eigenvector angle
ECG: Electrocardiogram
FA: Fractional anisotropy
FT: Feature tracking
GCS: Global longitudinal strain
GLS: Global longitudinal strain
GRS: Global radial strain
HA: Helix angle
LAD: Left anterior descending coronary artery
LCX: Left circumflex coronary artery
LGE: Late gadolinium enhancement
LHM: Left-handed myocytes
LV: Left ventricle/left ventricular
LVEDV: Left ventricular end-diastolic volume
LVEDVI: Left ventricular end-diastolic volume index
LVEF: Left ventricular ejection fraction
MD: Mean diffusivity
MI: Myocardial infarction
mrEF: Mid-range left ventricular ejection fraction
MOLLI: Modified Look-Locker inversion recovery
MVO: Microvascular obstruction
PCI: Percutaneous coronary intervention
pEF: Preserved left ventricular ejection fraction
RCA: Right coronary artery
RHM: Right-handed myocytes
rEF: Reduced left ventricular ejection fraction
ROI: Regions-of-interest
RV: Right ventricle/right ventricular
SCS: Segmental circumferential strain
SENSE: Sensitivity encoding
SE: Spin echo
SLS: Segmental longitudinal strain
SRS: Segmental radial strain
STEAM: Single -shot stimulated echo acquisition mode
STEMI: ST elevation myocardial infraction
